# Maternal mental well-being and recent child illnesses–A cross-sectional survey analysis from Jigawa State, Nigeria

**DOI:** 10.1371/journal.pgph.0001462

**Published:** 2023-03-02

**Authors:** Julius Salako, Damola Bakare, Tim Colbourn, Adamu Isah, Osebi Adams, Funmilayo Shittu, Obioma Uchendu, Ayobami A. Bakare, Hamish Graham, Eric D. McCollum, Adegoke G. Falade, the INSPIRING Consortium, Rochelle A. Burgess, Carina King

**Affiliations:** 1 Department of Health Promotion and Education, University College Hospital, Ibadan, Nigeria; 2 Department of Epidemiology and Medical Statistics, University College Hospital, Ibadan, Nigeria; 3 Institute for Global Health, University College London, London, United Kingdom; 4 Save the Children International, Abuja, Nigeria; 5 Department of Global Public Health, Karolinska Institutet, Stockholm, Sweden; 6 Department of Community Medicine, University College Hospital, Ibadan, Nigeria; 7 Department of Community Medicine, University of Ibadan, Ibadan, Nigeria; 8 Centre for International Child Health, Murdoch Children’s Research Institute, University of Melbourne, Royal Children’s Hospital, Parkville, Victoria, Australia; 9 Eudowood Division of Pediatric Respiratory Sciences, Department of Pediatrics, School of Medicine, Johns Hopkins University, Baltimore, MD, United States of America; 10 Department of Paediatrics, University of Ibadan, Ibadan, Nigeria; 11 Department of Paediatrics, University College Hospital, Ibadan, Nigeria; University of Regina, CANADA

## Abstract

Child health indicators in Northern Nigeria remain low. The bidirectional association between child health and maternal well-being is also poorly understood. We aim to describe the association between recent child illness, socio-demographic factors and maternal mental well-being in Jigawa State, Nigeria. We analysed a cross-sectional household survey conducted in Kiyawa local government area, Jigawa State, from January 2020 to March 2020 amongst women aged 16–49 with at least one child under-5 years. We used two-stage random sampling. First, we used systematic random sampling of compounds, with the number of compounds based on the size of the community. The second stage used simple random sampling to select one eligible woman per compound. Mental well-being was assessed using the Short Warwick-Edinburgh Mental Wellbeing Score (SWEMWBS). We used linear regression to estimate associations between recent child illness, care-seeking and socio-demographic factors, and mental well-being. Overall 1,661 eligible women were surveyed, and 8.5% had high mental well-being (metric score of 25.0–35.0) and 29.5% had low mental well-being (metric score of 7.0–17.9). Increasing wealth quintile (adj coeff: 1.53; 95% CI: 0.91–2.15) not being a subsistence farmer (highest adj coeff: 3.23; 95% CI: 2.31–4.15) and having a sick child in the last 2-weeks (adj coeff: 1.25; 95% CI: 0.73–1.77) were significantly associated with higher mental well-being. Higher levels of education and increasing woman’s age were significantly associated with lower mental well-being. Findings contradicted our working hypothesis that a recently sick child would be associated with lower mental well-being. We were surprised that education and late marriage, which are commonly attributed to women’s empowerment and autonomy, were not linked to better well-being here. Future work could focus on locally defined tools to measure well-being reflecting the norms and values of communities, ensuring solutions that are culturally acceptable and desirable to women with low mental well-being are initiated.

## Background

⁠According to World Health Organisation (WHO) estimates, Sub-Saharan Africa and Central and Southern Asia accounted for more than 80% of the 5.2 million under-five deaths in 2019 [1]. Pneumonia, malaria and diarrhoea accounted for 29% of global deaths among children under 5 in 2018 [2]. While deaths from these causes have reduced since 2000 in the African region, hotspots of high mortality remain, with pneumonia accounting for 15% of under-five deaths in Nigeria, with more than 2,500 deaths per day [3]. Specifically, northern Nigeria has been identified as a regional hotspot for pneumonia deaths [4].

The risk of mortality in under-five children is associated with several socio-demographic factors, including the mother’s emotional well-being and survival, sanitation, and the socio-economic status of their household [5]. Nigeria ranks among the top ten countries globally for maternal deaths [6], with the weak health system identified as a key underlying cause as women face barriers in accessing care before, during and after pregnancy [7]. Moucheraud et al. reported that children of a mother who died within 42 days of their birth faced a 46-times greater risk of dying within one month when compared to children whose mothers survived [8, 9].

The prevalence of maternal postpartum depression is also high in Nigeria, with prevalences of 22.9% and 35.6% reported amongst mothers attending postpartum clinics in Enugu [10] and Lagos states [11], respectively. In many cultures, mothers are the main custodian of their child’s health [12]. In a facility-based study from Lagos, 64% of interviewed mothers were aware of the importance of key pillars of child well-being, such as complementary foods being introduced at 6 months, and 85% were aware of the basic practices to support healthy child growth, such as exclusive breastfeeding, proper sanitation and clothing [13]. However, Adewuya et al. found that depressed mothers were more likely to stop breastfeeding earlier and their infants more likely to have episodes of diarrhoea and other common infections [14].

Women’s mental well-being, and their ability to care for their children, can be influenced by the household they belong to [15, 16]. Common household factors that can affect health include rearing of animals in the house, use of unclean cooking fuels, family type and household decision-making processes. The quality of family relationships, including emotional support and resource availability, can influence a child’s well-being through psychosocial, behavioural and physiological pathways. For example, domestic violence, household food insecurity and poor health care practices can put under-five children at a greater risk of morbidity and mortality [15, 17, 18]. In rural Northern Nigeria, it is common for extended families to live in shared compounds, with an element of shared resources [19]. Additionally, 44% and 47% of women aged 15–49 in the Northeast and Northwest regions of Nigeria were in polygamous unions in 2017 [20]. A study by Akinyemi et al. [2016] found the risk of death in childhood was 23% higher in extended family households compared to nuclear family households [15]. Early marriage also remains a common practice in Northern Nigeria [21, 22] and has clear relationships to poor mental health outcomes [23]. Given gendered roles within households, women’s autonomy in decision making about the health of themselves and their children is low, with men considered as the final authority within the family [24–27]. Women are therefore highly dependent on their husbands in terms of decision making, including decisions relating to their own and their children’s health [28].

Despite the launch of initiatives to reduce child mortality and improve maternal health in this region, little is known about the multi-directional interactions between women’s well-being and acute child illness [15, 18, 29]. Therefore, we aim to describe the association between recent childhood illness and maternal mental well-being in Jigawa state, northern Nigeria.

## Methodology

We conducted a secondary analysis of cross-sectional data from women with children under the age of five, residing in Kiyawa Local Government Area (LGA), Jigawa State, Nigeria. Data were collected as part of the wider baseline survey for the INSPIRING Project (trial registration: ISRCTN39213655) [30] from January 7^th^ 2020 to March 17^th^ 2020. On this date, all field activities were paused as a precaution due to the COVID-19 pandemic, however no cases had been recorded in Jigawa State at that time.

### Setting

Kiyawa LGA consists of 3 districts: Kiyawa district, Shuwarin district and Abalago district, with a total of 11 wards, which are the administrative units used for electoral purposes. Kiyawa LGA had a population of 172,952 in the last census conducted in 2006 and is currently estimated to be 230,000 [30, 31]. The socio-cultural situation in the state is homogeneous and mostly populated by the Hausa-Fulani; over 99% of the people in Kiyawa practice Islam and it is predominantly rural with agriculture as the main occupation [30, 32, 33].

### Study population

The study is limited to women of childbearing age between, 16–49 years old, who are permanent residents of Kiyawa LGA. We interviewed women who had at least one child under-five years at the time of the study visit. All villages within Kiyawa LGA were included in the survey.

### Sampling and recruitment

Women were sampled using a two-stage random sampling strategy. First, given that villages in this setting are often formed of dispersed compounds, we considered a group of compounds that identified under the same village name as a community. Data collectors, with the aid of a local gatekeeper (e.g. village head), located the “centre” of the village. Using an Expanded Programme of Immunisation [EPI] sampling method, the data collectors randomly selected where to start numbering the compounds by spinning a bottle on the ground. The compounds were numbered sequentially in clockwise order, going from the centre of the village outwards in a spiral and marking each compound with chalk or marker pens. For villages where it was difficult to identify a centre, the study mapper together with local gatekeepers identified the first compound at the entrance of the village and mapped quadrants in a clockwise pattern till all quadrants were completed.

Compounds were then sampled using systematic random sampling proportional to size, with an average sampling interval of 10, depending on the total size of the community. The CommCare software used for data collection randomly generated the number of the first compound and calculated the sampling interval and the total number of compounds to be sampled. A minimum of three compounds were sampled in all communities, meaning some villages are over-represented in the data. If a sampled compound was not eligible or available [e.g. no eligible women resided there], the data collectors moved to the immediate neighbour as a replacement. Within each compound, all eligible women were assigned a sequential number and sampled using simple random sampling within the CommCare tool using a random number generator.

### Data collection

#### Data collection procedure

We recruited and trained 36 data collectors and 2 field supervisors. Data collectors worked in 12 teams, with each team consisting of a male community mapper, a clinical data collector and a female non-clinical data collector. The non-clinical data collectors were responsible for conducting interviews with women and heads of compounds. All the data collectors had one-week training, with 3 days of in-class training, and 3 days’ field training where data collectors watched experts conduct the woman interview and thereafter simulated the entire process.

A non-clinical data collector administered a questionnaire to any member of the compound who was generally recognized by other compound members as the ‘head’, irrespective of age and gender. Questions on personal information and assets were asked in private, as the interviewed person might not want to share this information with the other people living in the compound. Questions on compound structure, compound members and their interpersonal relations were jointly answered by the compound head and other compound members. A separate woman questionnaire was administered to the randomly selected woman. The data collector asked the last time their child fell sick and whether they sought care for all their children under five, as well as questions on socio-demographics and mental well-being. We used the Short Warwick-Edinburgh Mental Wellbeing Scale (SWEMWBS) to assess mental well-being, which includes 7 questions [34]. Interviews with women were conducted privately, due to the sensitivity of some questions.

#### Validity and reliability of instrument

We piloted the data collection tools to ensure relevance, appropriateness and adequacy of the items among residents of Kaci and Kudai communities, which are 43km away from Kiyawa LGA. Residents of Kaci and Kudai were recruited for the pre-test because of their similarity in socio-demographic characteristics with the people of Kiyawa. Questions were translated from English to Hausa. The translation of the SWEMWBS to Hausa was coordinated by three members of the research team: a community psychologist and mental health expert [RAB], qualitative fieldwork manager and Hausa speaker [FS], and medical practitioner [AAB]. Through iterative discussion a translated tool was developed, re-tested in a focus group and back-translated by a Hausa speaker, independent of the research team, to ensure the translated items maintained their internal reliability.

### Data analysis

We described compound and woman characteristics using proportions and means and summarized the SWEMWBS score. An overall SWEMWBS score was generated by first summing the scores for each of the seven items, which are scored from 1 to 5, to give a raw score (range: 7–35). The total raw score was then transformed into metric scores as recommended for comparability using the SWEMWBS conversion table [35], still with a range of 7–35. We generated a categorical variable for mental well-being, with <18 points indicating low well-being, 18–25 moderate well-being and >25 high well-being, with the low well-being cut-point of <18 being correlated with probable depression according to short warwick classification [36]. Statistical associations between socio-demographic factors and the categorical well-being variables were estimated using chi-2 and Fisher’s exact tests.

However, given SWEMWBS scores were normally distributed, we estimated the association between recent child illness and care-seeking and mental well-being using linear regression, using the continuous metric SWEMWBS score as the outcome for comparability. We selected the following potential confounders a priori, based on published literature: woman’s age, age at first marriage, occupation, education, wealth, ranking (the order of marriage) amongst their husband’s wives and the number of children. The regression model was adjusted for those variables which had a p-value<0.1 in bivariate analysis. As missing data was uncommon, we conducted a complete case analysis.

### Ethics

Ethical approval was obtained from Jigawa State Government (ref: JPHCDA/ADM/GEN/073/V.I) and the University College London Research Ethics Committee (ref: 3433/004). Approval was obtained from the Local Government, District, Ward and Village head of all communities before data collection commenced in any community. Consent was sought verbally from all participants after going through the informed consent form. They were informed that participation is voluntary and that the data collected would be used for research purposes only.

## Results

### Participant description

**Fig 1:**
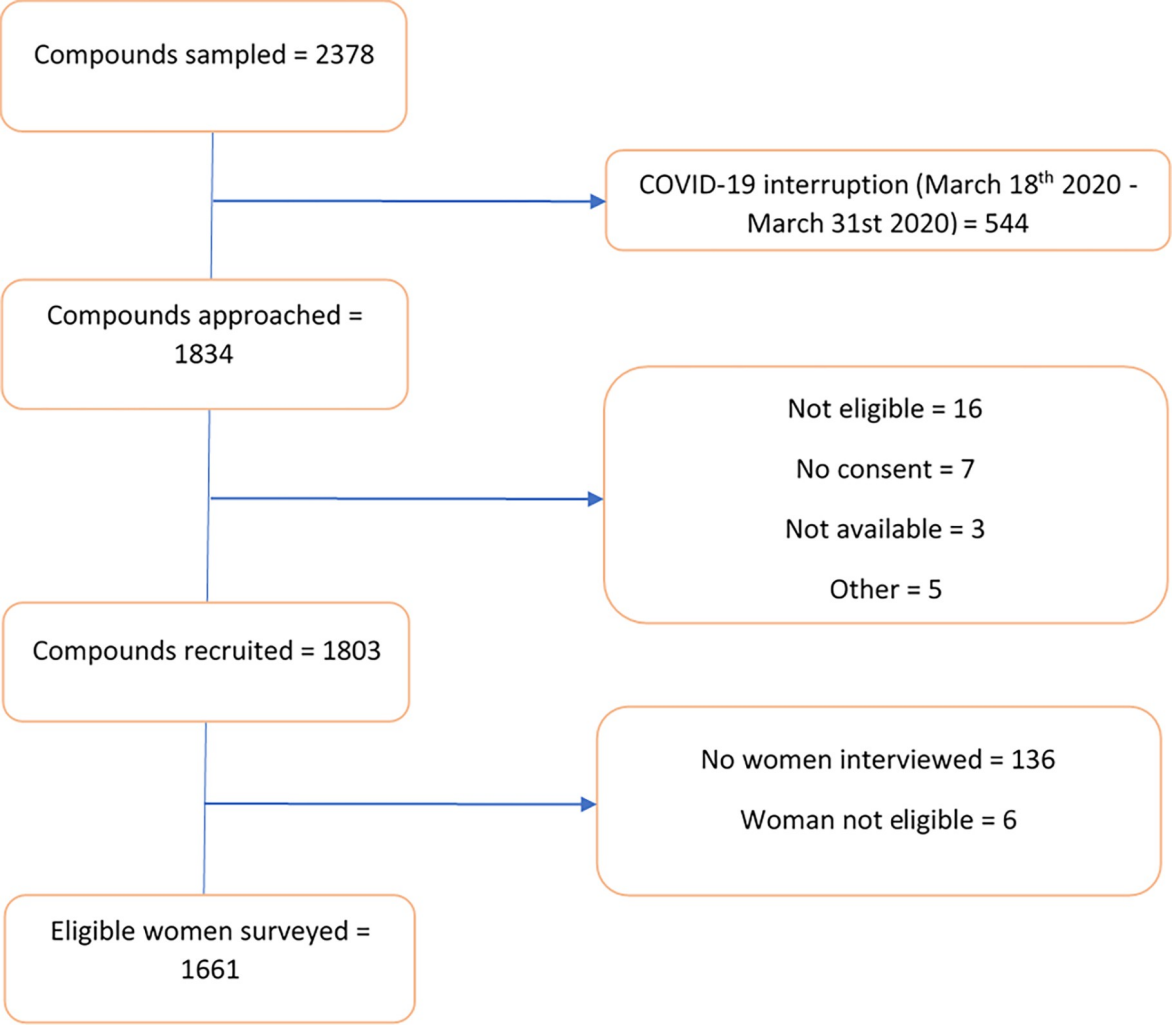
Participant inclusion flow diagram.

Overall, we recruited 1,661 eligible women, amongst the 2,378 compounds that were originally sampled (Fig 1). A summary of compound and woman characteristics are presented in Table 1. Overall, 64.0% of the compound heads had an informal or religious education, with 55.6% of them practising subsistence farming as their main source of livelihood. The mean age and age at first marriage of women recruited for the study was 28.9 years and 15.1 years, respectively, and the mean number of surviving children of any age and number of surviving children under-five years was 3.6 and 1.6 children, respectively. A quarter (25.3%) of the women reported that their child was sick in the two weeks before the survey, and 12.8% had sought care for their child in the last two weeks.

**Table 1:** Socio-economic and demographic status of women and compound head (n = 1661).

Compound variables (n, %)		N	%
Head of Compound age	20–29	29	1.7
	30–39	250	15.1
	40–49	672	40.1
	50–59	374	22.9
	60–69	224	13.5
	70 and above	112	6.7
Education of compound head*	No formal education	275	16.7
	Informal/religious education	1061	64.0
	Primary	100	6.0
	Secondary	120	7.2
	Tertiary/further	102	6.1
Occupation of compound head	Subsistence farming	924	55.6
	Unskilled manual labour	119	7.2
	Skilled manual labour	177	10.7
	Small business owner	227	13.7
	Professional	87	5.2
	Traditional healer/Imam	41	2.4
	Student/unemployed/retired	86	5.2
Women aged 16–49 with under-5 child per compound	1	679	40.9
	2	457	27.5
	3	229	13.8
	4	140	8.4
	5	68	4.1
	6 and above	88	5.3
Wealth quintile	Lowest	355	21.4
	Low	311	18.7
	Middle	359	21.6
	High	305	18.4
	Highest	331	19.9
Woman variables		N	%
Woman’s age	16–19	100	6.0
	20–29	781	47.0
	30–39	620	37.3
	40–49	160	9.7
Age at first marriage**	<15	539	32.4
	15–17	980	59.4
	>17	136	8.2
Woman’s ranking amongst her husband’s wives ***	1	1311	79.6
	2	259	15.8
	3	59	3.6
	4	17	1.0
Number of surviving children of any age	1	248	15.0
	2	353	21.2
	3–5	756	45.5
	> = 6	304	18.3
Number of surviving children under-five years****	1	783	47.4
	2	751	45.3
	3 and 4	121	7.3
Women whose child was sick in last 2 weeks	No	1241	74.7
	Yes	420	25.3
Women who sought care for a child	No	1448	87.2
	Yes	213	12.8

* Values recorded as “don’t know” were set to missing (n = 3)

** Women who did not report their age at marriage were set to missing (n = 6)

*** Values of more than 4 were set to missing as the maximum number of wives permitted within Islam is 4 (n = 15)

**** Women reporting more than 4 surviving children under-five were set to missing (n = 6)

### SWEMWBS scores

Using the SWEMWBS classification for women’s well-being, 8.5% had high mental well-being, 62.0% moderate, and 29.5% had low mental well-being–Table 2. The scores ranged across the whole spectrum from 7–35, with a mean value of 19.3 and a standard deviation of 4.1, and there was some variation between individual well-being questions (S1 Appendix). There was a significant difference in mental well-being according to wealth quintile, with a positive trend for those in the highest wealth quantile (p-value <0.001). Very few women (0.7%) who practice subsistence farming had high mental well-being compared to 54.6% who are small business owners and professionals (p-value<0.001). Women who reported that their child was sick in the last 2-weeks had higher mental well-being compared to those that did not report a sick child within the same period. Of all the women who reported that their child was sick in the last 2-weeks, 36.9% had high mental well-being compared to 15.9% who had low mental well-being (p-value<0.001).

**Table 2:** Summary of well-being score, according to key compound and woman characteristics.

Variables		Metric Low score (*7*.*0–17*.*9)*		Metric Medium *score (18*.*0–24*.*9)*		Metric High *score (25*.*0–35*.*0)*		p-value
		N	(%)	N	(%)	N	(%)	
Total		490	(29.5%)	1030	(62.0%)	141	(8.5%)	
Number of children	1	68	(13.9%)	160	(15.5%)	20	(14.2%)	0.703
	2	105	(21.4%)	224	(21.8%)	24	(17.0%)	
	3–5	228	(46.5%)	462	(44.9%)	66	(46.9%)	
	6 and above	89	(18.2%)	184	(17.9%)	31	(22.0%)	
Number of under-five children	1	219	(44.7%)	504	(49.0%)	60	(42.6%)	0.132
	2	237	(48.4%)	447	(43.4%)	67	(47.5%)	
	3 and 4	34	(6.9%)	74	(7.2%)	13	(9.2%)	
First Marriage	No	45	(9.2%)	105	(10.2%)	28	(19.9%)	0.001
	Yes	445	(90.8%)	925	(89.9%)	113	(80.1%)	
Age at first marriage*	Less than 15	139	(28.3%)	341	(33.1%)	59	(41.8%)	0.029
	15–17	309	(63.1%)	604	(59.0%)	67	(49.0%)	
	Above 17	42	(8.6%)	81	(7.9%)	13	(9.2%)	
Woman’s ranking amongst her husband’s wives**	1	379	(77.9%)	835	(81.8%)	97	(70.2%)	0.018
	2	83	(17.0%)	143	(14.1%)	33	(23.4%)	
	3	17	(3.5%)	34	(3.3%)	8	(5.7%)	
	4	8	(1.6%)	8	(0.8%)	1	(0.7%)	
Woman’s main occupation	Subsistence farming	36	(7.4%)	47	(4.6%)	1	(0.7%)	<0.001
	Unskilled manual labour	76	(15.5%)	232	(22.5%)	35	(24.9%)	
	Skilled manual labour/ TBA	126	(25.7%)	106	(10.3%)	18	(12.8%)	
	Small business owner/Professional	235	(48.0%)	483	(46.9%)	77	(54.6%)	
	Not working	17	(3.5%)	162	(15.7%)	10	(7.1%)	
Woman’s age	16–19	17	(3.5%)	74	(7.2%)	9	(6.4%)	0.004
	20–29	222	(45.3%)	501	(48.6%)	58	(41.1%)	
	30–39	211	(43.0%)	352	(34.2%)	57	(40.4%)	
	40 and above	40	(8.2%)	103	(10.0%)	17	(12.1%)	
Woman’s highest Education***	No formal education	80	(16.3%)	375	(36.4%)	66	(46.9%)	<0.001
	Religious education	341	(69.6%)	514	(49.9%)	55	(39.0%)	
	Primary education	33	(6.7%)	83	(8.1%)	13	(9.2%)	
	Secondary and tertiary	29	(5.9%)	57	(5.5%)	6	(4.3%)	
Wealth Quintile	Lowest	110	(22.5%)	230	(22.3%)	15	(10.6%)	<0.001
	Low	93	(19.0%)	194	(18.9%)	24	(17.0%)	
	Middle	117	(23.9%)	221	(21.5%)	21	(14.9%)	
	High	81	(16.5%)	192	(18.7%)	32	(22.7%)	
	Highest	89	(18.2%)	193	(18.7%)	49	(34.8%)	
Child was sick in last 2-weeks	No	412	(84.1%)	740	(71.8%)	89	(63.1%)	<0.001
	Yes	78	(15.9%)	290	(28.2%)	52	(36.9%)	
Sought care for child in last 2-weeks	No	442	(90.2%)	895	(86.9%)	111	(78.7%)	0.001
	Yes	48	(9.8%)	135	(13.1%)	30	(21.3%)	

P-values were calculated using the chi-square test unless otherwise indicated.

*Women who did not report their age at marriage were set to missing (n = 6)

**P-value calculated using Fisher’s exact test. Values of more than 4 were set to missing as the maximum number of wives permitted within Islam is 4 (n = 15).

***Women who did not report their highest education attained were set to missing (n = 9)

### Association between SWEMWBS and child illness

Table 3 shows the results of the adjusted linear regression model. Having a sick child in the last 2-weeks was significantly associated with a 1.25-point increase in women’s SWEMWBS scores (adj coeff: 1.25; 95% CI: 0.73, 1.77). Women in the higher wealth quintiles, and whose main occupation is not subsistence farming had a positive association with well-being. Being older and women with higher educational attainment were associated with lower well-being.

**Table 3:** Multivariable linear regression analysis of recent child illness and care-seeking and women’s well-being (adjusted R-squared = 0.159).

Variable		Adjusted coefficient	95% confidence interval		p-value
Child was sick in last 2-weeks	No	ref			
	Yes	1.25	(0.73	1.77)	<0.001
Sought care for a child in last 2-weeks	No	ref			
	Yes	0.01	(-0.67	0.69)	0.978
Wealth Quintile	Lowest socioeconomic status	ref			
	Low/Middle socioeconomic status	0.97	(0.39	1.55)	0.001
	Middle socioeconomic status	0.49	(-0.07	1.05)	0.084
	Middle/High socioeconomic status	1.13	(0.54	1.72)	<0.001
	Highest socioeconomic status	1.53	(0.91	2.15)	<0.001
Woman’s age	16–19	ref			
	20–29	-1.48	(-2.27	-0.69)	<0.001
	30–39	-1.60	(-2.40	-0.80)	<0.001
	40 and above	-0.96	(-1.91	0.02)	0.046
Woman’s age at first marriage	Less than 15	ref			
	15–17	-0.54	(-0.96	-0.12)	0.012
	Above 17	0.06	(-0.70	0.88)	0.822
Woman’s ranking amongst her husband’s wives	1	ref			
	2	-0.14	(-0.64	0.36)	0.586
	3	0.44	(-0.56	1.44)	0.386
	4	-1.21	(-3.02	0.59)	0.188
Woman’s main Occupation	Subsistence farming	ref			
	Unskilled manual labour	3.23	(2.31	4.15)	<0.001
	Skilled manual labour/TBA	0.69	(-0.25	1.63)	0.152
	Small business owner/Professional	2.80	(1.94	3.66)	<0.001
	Not working	2.88	(1.87	3.89)	<0.001
Education	No formal education	ref			
	Informal/Religious education	-1.65	(-2.10	-1.19)	<0.001
	Primary education	-1.29	(-2.05	-0.53)	0.001
	Secondary and tertiary	-2.32	(-3.28	-1.36)	<0.001

## Discussion

We examined the association of individual and household characteristics and recent childhood illness with woman’s mental well-being in Kiyawa LGA of Jigawa State, Nigeria. We found that increasing wealth quintile (adj coeff: 1.53; 95% CI: 0.91–2.15), a non-subsistence farming occupation (highest adj coeff: 3.23; 95% CI: 2.31–4.15) and having a sick child in the last 2 weeks (adj coeff: 1.25; 95% CI: 0.73–1.77) were positively associated with higher mental well-being, the latter result being unexpected. Higher levels of education and increasing age were linked with lower mental well-being, while woman’s age at first marriage had a mixed association, contrary to prior literature in this area. However, it should be noted, that while these associations were generally highly statistically significant, they correspond to relatively small changes in the SWEMWBS score and therefore the clinical and policy relevance may be minor.

The WHO defines mental health as a state of well-being where individuals can cope with the stresses of everyday life, realizes their capabilities and can contribute to their wider community [37]. With such a definition, the emphasis is on the importance of functionality in one’s life, which moves beyond the presence or absence of a mental disorder. While there is a wide range of tools that focus on the diagnosis of disease, fewer tools exist that attempt to grapple with the positive dimensions of mental health, focusing on the capacity and overall well-being [23, 38–40]. Even fewer have been widely used in sub-Saharan Africa [41, 42]. The importance of focusing on positive mental health is also crucial [43], in spaces where access to mental health services is limited, biomedical knowledge about mental conditions is low, and mental health stigma is high [44]. As such, we used the SWEMWBS tool with its 7 positively structured statements [45], which focuses on well-being instead of diagnosing a mental health condition and has been validated in a range of populations [38–40]. In addition, both the 7 and 14 item WEMWBS questions have been used in Nigeria previously [46–48], although we could not find a formal translation or validation within a Hausa-Fulani population.

Counter to our hypothesis, we observed that women who reported a child being sick in the 2-weeks before the survey had a small but significantly higher well-being than those who did not report a sick child. This contradicts prior literature conducted amongst caregivers of chronically ill children, where parents have been reported to experience deteriorating mental health [49–51]. A study from North-West Nigeria reported high rates of common mental health disorders amongst women of children attending out-patient malnutrition services [52]. However, chronic and acute illnesses will present different stressors, and there is a considerable literature gap on well-being responses to common childhood infections to compare our findings.

Within this context, a sick child often allows for the mobilization of resources, especially for women whose husbands control household finances and may withhold support [28]. Given the high child mortality [53], common child illnesses often have serious consequences and therefore their survival or recovery could lead to relief, as well as the immediate need for money providing an opportunity for a temporary increase in women’s financial status. It is also possible that child illnesses result in having more family and friends around you to offer support, something we’ve observed in this setting, and found elsewhere [54–57]. Both of these factors could explain a period of improved well-being during or immediately following acute child illness episodes. On the other hand, it may reflect reverse causality, where women’s well-being is acting on their ability to report a recent illness (i.e. women who have better well-being may be more able to recognize that their child was sick, and take action to improve their child’s health).

We found the higher the level of education a woman attains, the lower her reported mental well-being. This is in contrast to previous international [58–60] and local studies [61, 62], where education has been positively associated with better mental health; although not necessarily using the SWEMWBS tool for well-being assessment. The educational qualifications of women in Kiyawa LGA was very low, with only 13.4% having a minimum of primary education. This was then reflected in the occupations held, with 20.7% engaged in unskilled manual labour, and less than 5% reported being ‘professionals’. Jonathan et al. [63] provide a potential hypothesis for our observation, reporting that job and life satisfaction was lower amongst those highly educated since education leads to high aspirations. When these aspirations are not met, this can negatively affect mental well-being. Therefore, if access to education is not resulting in employment or other opportunities for women in this context, this might result in lower well-being. Even though our study did not capture domestic violence, more educated women may oppose some of the cultural norms that economically, socially and educationally neglect women [64–66]. The 2018 DHS reported 24% of women with primary education and 23% of those with secondary education experienced physical violence from their spouse, compared to 16% of women with no education [67]. However, evidence around this relationship is mixed and needs to be understood in the wider multi-directional context of social, economic and cultural factors [67–69].

Household wealth is one of the most significant predictors of health [61, 70–72]. Our results suggest that increased wealth (and the potential for resulting financial security, especially if there is competition for resources due to polygamous family structures) likely brought reassurance and therefore improved well-being. But the mechanism for achieving this amongst women was unlikely to have been through work and education–despite initiatives to empower women and improve their wealth often focussing on these. Therefore, initiatives in this region need to investigate locally defined solutions that would be culturally acceptable and desirable to women for poverty alleviation.

The association between a woman’s age at first marriage and their well-being was not clear in this study, despite hypothesizing that this may be an important factor given prior evidence that younger brides experience poorer mental health outcomes [73, 74]. The reasons for the previously reported negative associations include: being unable to negotiate safe sex, being at higher risk for early pregnancy, struggling to cope with leaving their family, and pressures of having to raise children before they are ready [75, 76]. However, in this context, getting married younger is the cultural norm with over 90% of women married before they were 17 years old. And so complying with these norms may help to make you feel fulfilled and not an inhibitor to other opportunities [23]. It’s also important to note that marriage can result in a better standard of life compared to the standard of living of their parental home [77].

We found a negative association between increasing age and mental well-being. Given that a woman’s age is linked to the number of years spent in marriage, a negative effect could be explained by the more years a woman spends in a marriage, the higher her chances of being in a polygamous marriage and therefore concerns over their status in the household may arise; however, a woman’s ranking amongst her husband’s wives was not significantly associated to well-being. Jigawa state is governed by Sharia law which allows a man to have more than one wife, and the 2018 DHS found that 44.7% of women in Jigawa state were in polygamous unions. Alean & John [78] found that women in polygamous marriages showed significantly higher psychological distress, and higher levels of anxiety, phobia and other psychological problems and they also had significantly more problems in family functioning, marital relationships and life satisfaction. Marriage is thought to protect well-being by providing companionship, emotional support, and economic security, but when the spouse is not supportive, this can take a turn on mental well-being [79].

Our study used a large representative community sample of women with children under-five in Kiyawa LGA, improving our generalizability for this population. However, we had four key limitations. Firstly, we did not complete the survey due to the COVID-19 pandemic. Given the stressors of COVID-19, and a subsequent cholera outbreak, recession, and flooding which have occurred since the survey, the state of well-being may be considerably different now. Secondly, child illness was solely based on the caregiver’s recall, and we did not triangulate with other data sources, which may have introduced a bias. Thirdly, we observed considerable pooling to the middle answer in responses to the SWEMWBS questions (S1 Appendix), which may indicate women were unwilling or unable to answer the well-being questions. This tool has been previously validated [39, 46], and we went through a rigorous piloting and translation process to ensure it would be understood, however, this highlights the importance of indigenous understandings of well-being instead of applying a Western perspective of well-being (i.e. even though this tool has been validated, it originates from a high-income context and so may not reflect the same values which we presume lead to higher levels of well-being). Given different norms and values, we need tools that reflect the values of the communities we are working with. Future work could focus on the development of locally defined tools for well-being, ensuring community perspectives are centred, as tools can be valid but inappropriate. And lastly, we did not collect information on the marital status of participating women, which could have provided more insight into their present marital status.

## Conclusion

In this study, we found that women who had a recent illness amongst one of their children did not have a measurable reduction in well-being, but rather had a small increase in their well-being. The role of acute child illnesses on mental health has not been well studied in this population, and given there were also unexpected associations observed with higher levels of education and age at first marriage compared with existing evidence, there is a need to reflect on how we conceptualise drivers of well-being in this context. Future research should explore the underlying mechanisms behind these associations, and whether these could be adopted and sustained in interventions to improve women’s well-being in this setting.

## Supporting information

S1 Appendix Responses to individual SWEMWBS questions, by child illness status. (DOCX)
