# Supplementary material for: Maternal mental well-being and recent child illnesses–A cross-sectional survey analysis from Jigawa State, Nigeria
Source: PLOS Glob Public Health. 2023 Mar 2;3(3):e0001462. doi: 10.1371/journal.pgph.0001462 (PMC10121178; doi:10.1371/journal.pgph.0001462)
Supplement: S1 Appendix — (DOCX) [file pgph.0001462.s001.docx]

**Appendix 1: Responses to individual SWEMWBS questions, by child illness status**

|  | **Child sick in the last 2 weeks** | | |
| --- | --- | --- | --- |
|  | No  N (%) | Yes  N (%) | Total  N (%) |
| **1. Feeling optimistic about the future** | | | |
| None of the time | 157 (12.7) | 30 (7.1) | 187 (11.3) |
| Rarely | 209 (16.8) | 59 (14.1) | 268 (16.1) |
| Some of the time | 682 (55.0) | 246 (58.6) | 928 (55.9) |
| Often | 184 (14.8) | 71 (17.9) | 255 (15.4) |
| All of the time | 9 (0.7) | 14 (3.3) | 23 (1.4) |
| **2. Feeling useful about the future** | | | |
| None of the time | 173 (13.9) | 17 (4.1) | 190 (11.4) |
| Rarely | 201 (16.2) | 70 (16.7) | 271 (16.3) |
| Some of the time | 588 (47.4) | 208 (49.5) | 796 (47.9) |
| Often | 240 (19.3) | 98 (23.3) | 338 (20.4) |
| All of the time | 39 (3.1) | 27 (6.4) | 66 (4.0) |
| **3. I have been feeling relaxed** | | | |
| None of the time | 176 (14.2) | 23 (5.5) | 199 (12.0) |
| Rarely | 161 (13.0) | 39 (9.3) | 200 (12.0) |
| Some of the time | 582 (46.9) | 224 (53.3) | 806 (48.5) |
| Often | 237 (19.1) | 92 (21.9) | 329 (19.8) |
| All of the time | 85 (6.9) | 42 (10.0) | 127 (7.7) |
| **4. I have been dealing with problems well** | | | |
| None of the time | 130 (10.5) | 14 (3.3) | 144 (8.7) |
| Rarely | 174 (14.0) | 46 (11.0) | 220 (13.2) |
| Some of the time | 641 (51.7) | 250 (59.5) | 891 (53.6) |
| Often | 241 (19.4) | 82 (19.5) | 323 (19.5) |
| All of the time | 55 (4.4) | 28 (6.7) | 83 (5.0) |
| **5. I have been thinking clearly** | | | |
| None of the time | 149 (12.0) | 14 (3.3) | 163 (9.8) |
| Rarely | 187 (15.1) | 42 (10.0) | 229 (13.8) |
| Some of the time | 593 (47.8) | 229 (54.5) | 822 (49.5) |
| Often | 220 (17.7) | 85 (20.2) | 305 (18.4) |
| All of the time | 92 (7.4) | 50 (11.9) | 142 (8.6) |
| **6. I have been feeling close to other people** | | | |
| None of the time | 160 (12.9) | 17 (4.0) | 177 (10.7) |
| Rarely | 177 (14.3) | 46 (11.0) | 223 (13.4) |
| Some of the time | 564 (45.5) | 236 (56.2) | 800 (48.2) |
| Often | 231 (18.6) | 79 (18.8) | 310 (18.7) |
| All of the time | 109 (8.8) | 42 (10.0) | 151 (9.1) |
| **7. I have been able to make up my own mind about things** | | | |
| None of the time | 146 (11.8) | 17 (4.0) | 163 (9.8) |
| Rarely | 180 (14.5) | 43 (10.2) | 223 (13.4) |
| Some of the time | 642 (51.7) | 247 (58.8) | 889 (53.5) |
| Often | 234 (18.9) | 82 (19.5) | 316 (19.0) |
| All of the time | 39 (3.1) | 31 (7.4) | 70 (4.2) |
